# Supplementary material for: Structural basis for DNA damage-induced phosphoregulation of MDM2 RING domain
Source: Nat Commun. 2020 Apr 29;11:2094. doi: 10.1038/s41467-020-15783-y (PMC7190642; doi:10.1038/s41467-020-15783-y)
Supplement: Supplementary file 1 — Supplementary Information [file 41467_2020_15783_MOESM1_ESM.pdf]

Supplementary Information

Structural basis for DNA damage-induced phosphoregulation of MDM2 RING domain

Helge M. Magnussen, Syed F. Ahmed, Gary. J. Sibbet, Ventzislava A. Hristova, Koji Nomura, Andreas K. Hock, Lewis J. Archibald, Andrew G. Jamieson, David Fushman, Karen H. Vousden, Allan M. Weissman and Danny T. Huang

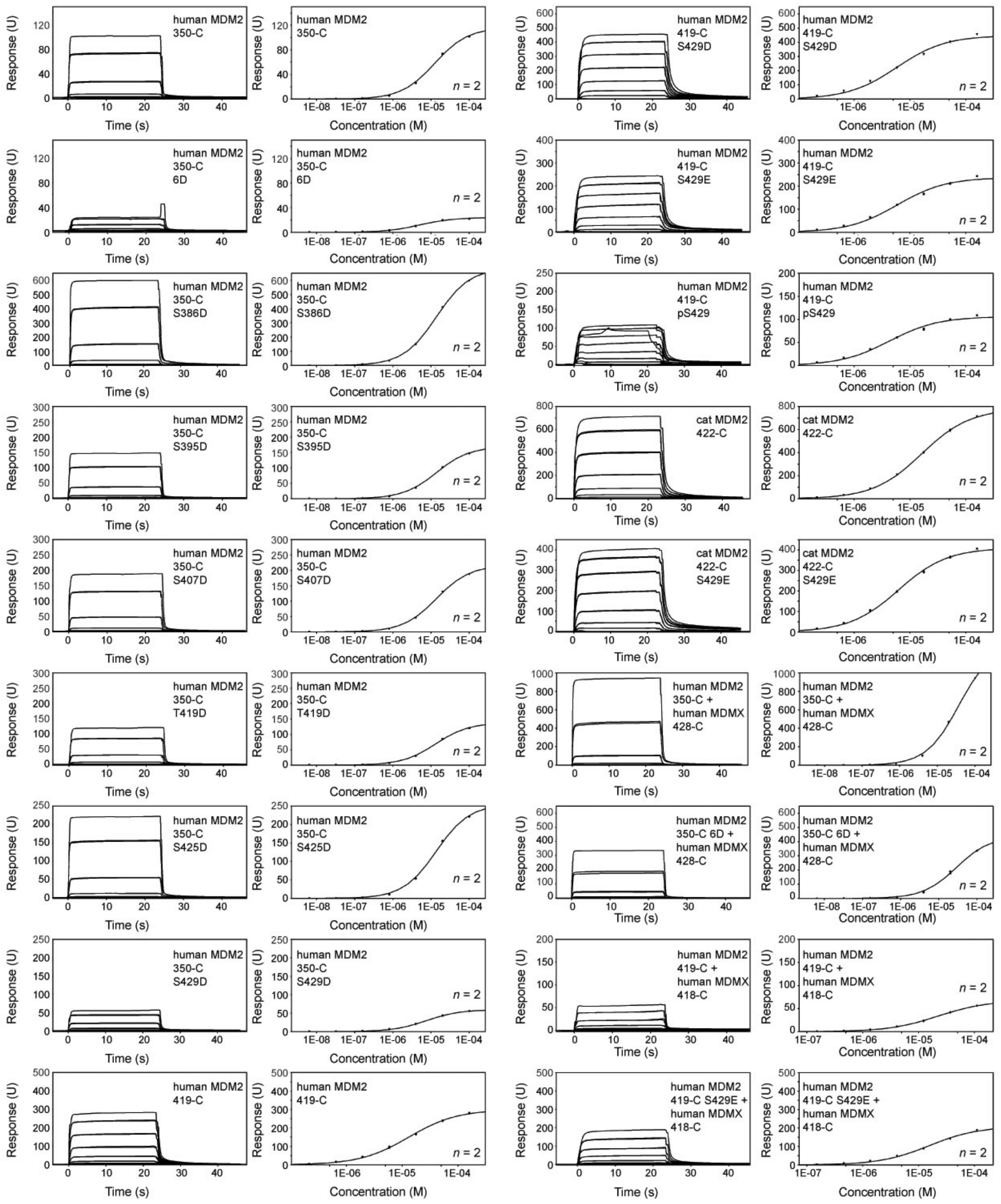

Supplementary Figure 1 SPR analyses of GST-MDM2 or GST-MDM2-MDMX variants and UbcH5B–Ub binding affinities, related to Table 1. Representative sensorgrams (left) and binding curves (right) for GST-MDM2 or GST-MDM2-MDMX variants (indicated) and UbcH5B–Ub. n=2 for each binding curve.

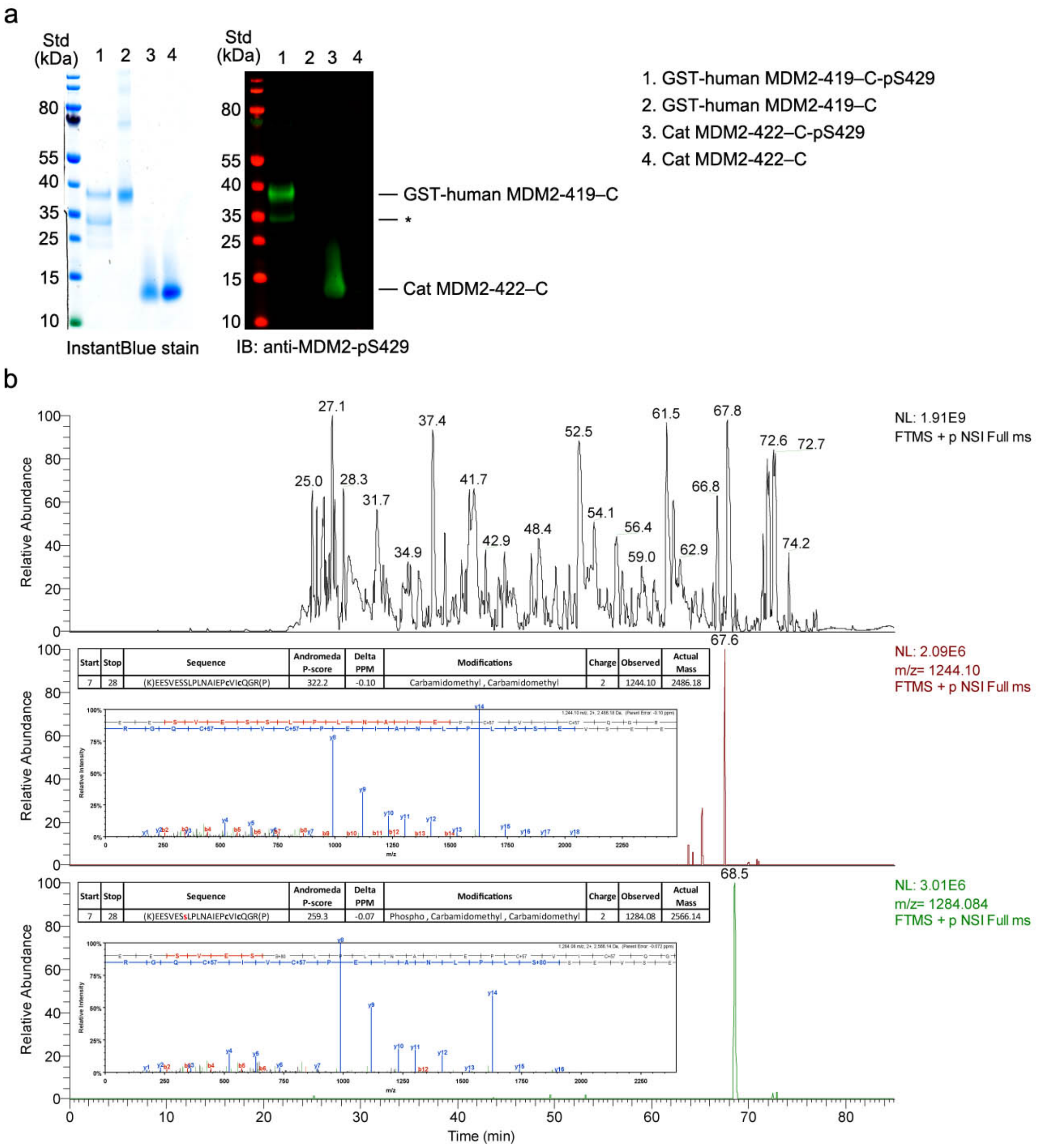

Supplementary Figure 2 Generation of S429 phosphorylated MDM2, related to Figure 4 and Table 1. (**a**) SDS-PAGE (left panel) and western blot (right panel) showing MDM2 variants detected with InstantBlue staining and anti-MDM2-pS429 antibody, respectively. MDM2-pS429 variants from human and cat were generated using the translational insertion of *O*-phosphoserine system. GST-human MDM2-419–C variants were purified by glutathione sepharose-affinity chromatography without further purification. Cat MDM2-422–C variants were purified by glutathione sepharose-affinity chromatography followed by TEV cleavage to remove the GST-tag and further purified by gel filtration

chromatography. Asterisk indicates a truncated GST-MDM2-419–C-pS429 product. The gels show that only MDM2 variants generated using the translational insertion of *O*-phosphoserine system reacted with our customized anti-MDM2-pS429 antibody, thereby confirming antibody specificity. The experiments were performed in triplicates with similar results. (**b**) Mass spectrometry analysis of GST-MDM2-419–C-pS429 from **a**. Top panel, chromatogram showing the tryptic peptides of GST-MDM2-419–C-pS429 separated by nanoscale C18 reverse-phase liquid chromatography using an EASY-nLC 1200 coupled online to an Orbitrap Q-Exactive HF mass spectrometer via nanoelectrospray ion source. Middle panel, MS/MS spectrum of a peak at 67.6 min shows the presence of unphosphorylated MDM2 peptide (m/z 1244.10). Bottom panel, MS/MS spectrum of a peak at 68.5 min shows the presence of S429-phosphorylated peptide (m/z 1284.08).

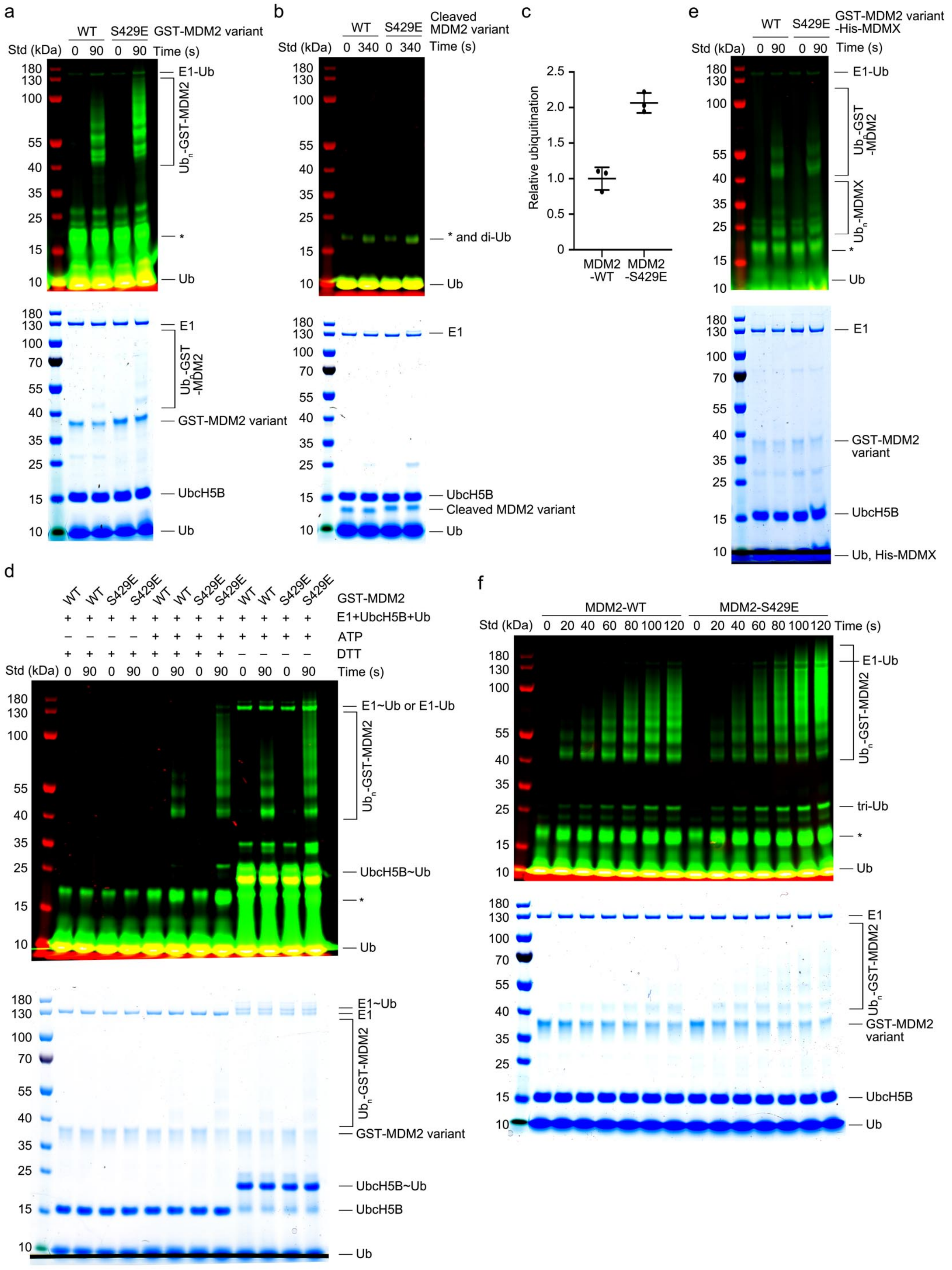

Supplementary Figure 3 S429E substitution enhances the E3 activity of MDM2 homodimer, related to Figure 1. (**a**) Uncropped reduced SDS-PAGE showing autoubiquitination reactions catalyzed by GST-MDM2-419–C and its S429E substitution

using fluorescently-labeled Ub and visualized with an Odyssey CLx Imaging System (top panel) and InstantBlue staining (bottom panel), related to **Figure 1a**. (**b**) Reduced SDS-PAGE showing ubiquitination reactions catalyzed by cleaved MDM2-419–C and its S429E substitution using fluorescently-labeled Ub and visualized with an Odyssey CLx Imaging System (top panel) and InstantBlue staining (bottom panel). Three independent experiments were performed with similar results. (**c**) Plot showing relative di-Ub formation corresponding to **b**. Data are presented as mean value ± SD from three independent experiments (n=3). Cleaved MDM2-419–C variants lack accessible lysine sites for ubiquitination and therefore utilize Ub as an acceptor to produce di-Ub. MDM2-S429E displayed enhanced activity as compared to MDM2-WT. (**d**) SDS-PAGE showing autoubiquitination reaction catalyzed by GST-MDM2-419–C and its S429E substitution using fluorescently-labeled Ub in the absence and presence of ATP and DTT, visualized with an Odyssey CLx Imaging System (top panel) and InstantBlue staining (bottom panel). The experiments were performed in triplicates with similar results. MDM2 autoubiquitination is dependent on the presence of ATP. UbcH5B~Ub remained charged throughout the duration of the reaction. (**e**) Uncropped reduced SDS-PAGE showing autoubiquitination reactions catalyzed by GST-MDM2-419–C-His-MDMX-418–C and its MDM2-S429E substitution using fluorescently-labeled Ub and visualized with an Odyssey CLx Imaging System (top panel) and InstantBlue staining (bottom panel), related to **Figure 1c**. (**f**) Uncropped reduced SDS-PAGE showing autoubiquitination reactions catalyzed by GST-MDM2-419–C and the S429E substitution over the indicated times using fluorescently-labeled Ub and visualized with an Odyssey CLx Imaging System (top panel) and InstantBlue staining (bottom panel), related to **Figure 1e**. Asterisks indicate trace background GGSC-Ub that disulfide-bonded via the engineered Cys and reacted non-specifically with IRDye® 800CW maleimide. This band overlapped with di-Ub in **b**.

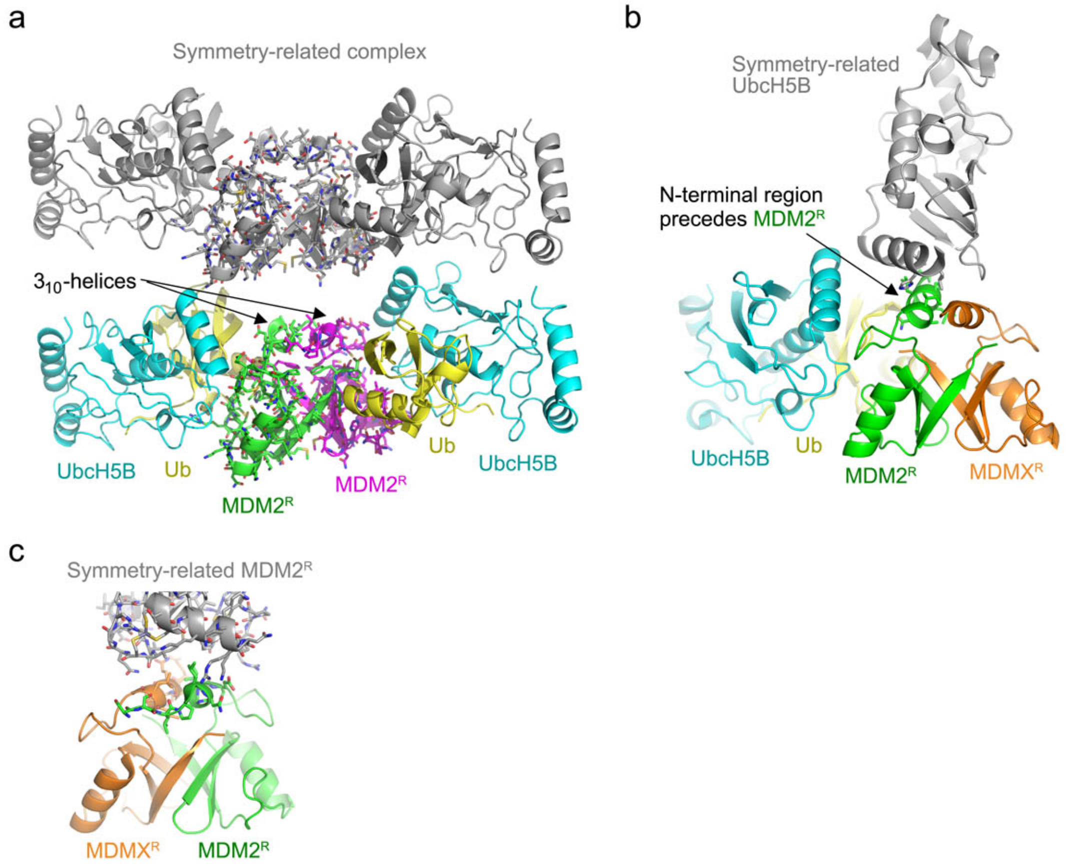

Supplementary Figure 4 Comparison of crystal packing in the structures of human MDM2-419–C-UbcH5B–Ub (a), human MDM2-428–C-MDMX-428–C-UbcH5B–Ub (b; PDB 5MNJ) and human MDM2-428–C-MDMX-428–C (c; PDB 2VJF) complexes related to Figure 3. (**a**) The N-terminal region preceding the MDM2 RING domain in the structure of human MDM2-419–C-UbcH5B–Ub complex is not involved in the crystal packing suggesting that symmetry-related molecules do not influence the 3_{10}-helical configuration. (**b,c**) The N-terminal region preceding the MDM2 RING domain in the structures of human MDM2-428–C-MDMX-428–C-UbcH5B–Ub and human MDM2-428–C-MDMX-428–C contacts a symmetry-related molecule. Crystal-packing contacts could contribute to the structural configuration observed for the N-terminal region preceding the MDM2 RING domain in the heterodimer. All coloring is as described in **Figure 2**. Symmetry-related molecules are in gray.

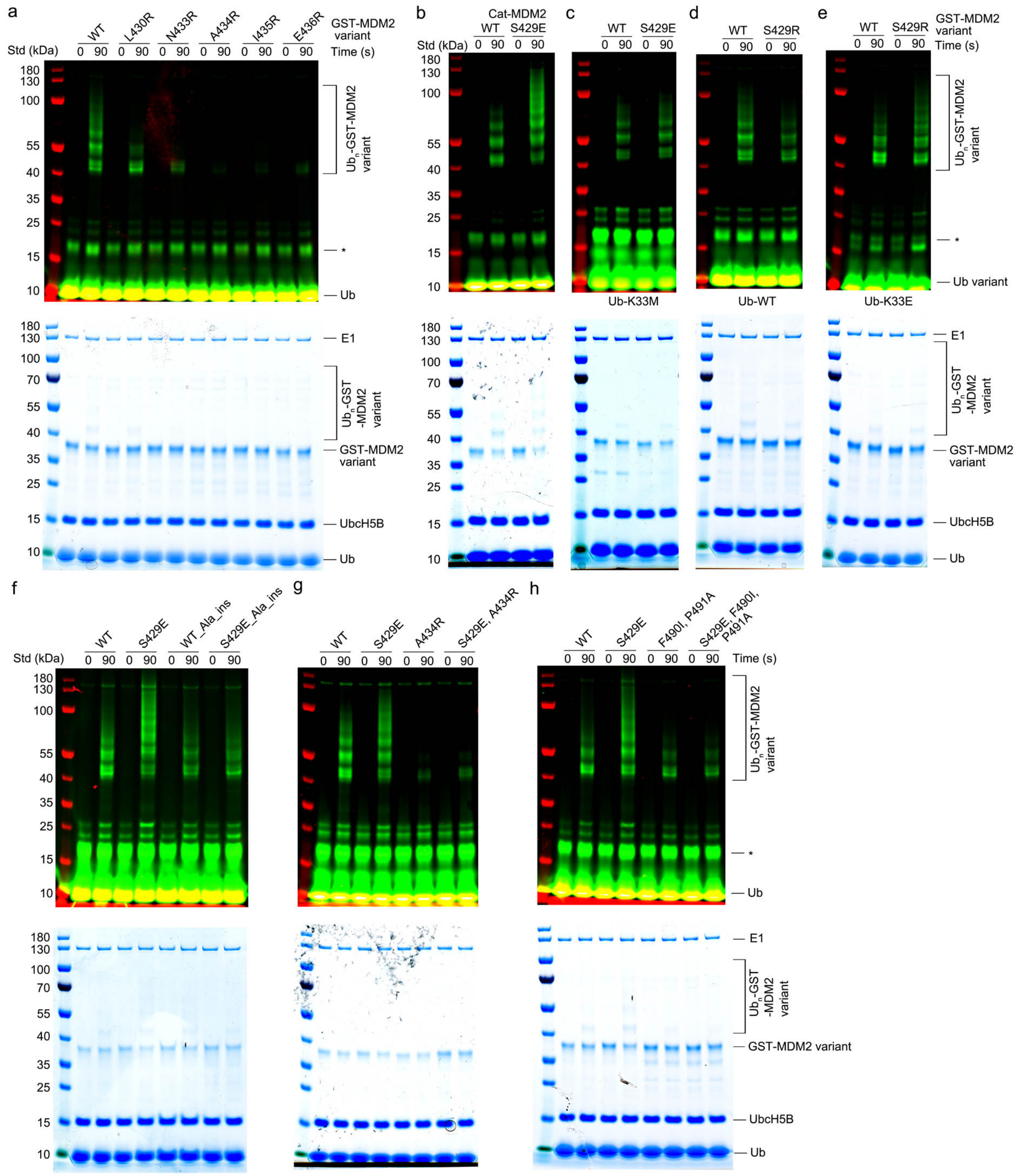

Supplementary Figure 5 Uncropped SDS-PAGE showing the ubiquitination activity MDM2 variants related to Figures 3–5. (**a**) Uncropped reduced SDS-PAGE showing autoubiquitination reactions catalyzed by GST-MDM2-419–C and variants using fluorescently-labeled Ub and visualized with an Odyssey CLx Imaging System (top panel) and InstantBlue staining (bottom panel), related to **Figure 3f**. (**b–e**) Uncropped reduced SDS-PAGE showing autoubiquitination reactions catalyzed by GST-MDM2 variants using

fluorescently-labeled Ub variant and visualized with an Odyssey CLx Imaging System (top panel) and InstantBlue staining (bottom panel), related to **Figures 4b, 4i, 4k and 4m**, respectively. (**f–h**) Uncropped reduced SDS-PAGE showing autoubiquitination reactions catalyzed by GST-MDM2 variants using fluorescently-labeled Ub and visualized with an Odyssey CLx Imaging System (top panel) and InstantBlue staining (bottom panel), related to **Figures 5f, 5h and 5j**, respectively. Asterisks indicate trace background GGSC-Ub that disulfide-bonded via the engineered Cys and reacted non-specifically with IRDye® 800CW maleimide.

	423 429	RING domain (→ 491)
**Mammalians**		
H. sapiens (423-C)	EESVES**S**LPLNAIE	PCVICQGRPKNGCIVHGKTGHLMACFTCAKKLKKRNKPCPVCRQPIQMIVLTYFP
L. vexillifer	EESVES**S**FPLNAIE	PCVICQGRPKNGCIVHGKTGHLMACFTCAKKLKKRNKPCPVCRQPIQMIVLTYFP
P. abelii	EESMES**S**LPLNAIE	PCVICQGRPKNGCIVHGKTGHLMACFTCAKKLKKRNKPCPVCRQPIQMIVLTYFP
C. porcellus	EESVEF**S**LPLNAIE	PCVICQGRPKNGCIVHGKTGHLMSCFTCAKKLKKRNKPCPVCRQPIQMIVLTYFP
S. scrofa	EESAES**S**FPLNAIE	PCVICQGRPKNGCIVHGKTGHLMACFTCAKKLKKRNKPCPVCRQPIQMIVLTYFP
O. aries	EESMES**S**FPLNAIE	PCVICQGRPKNGCIVHGKTGHLMACFTCAKKLKKRNKPCPVCRQPIQMIVLTYFP
C. lupus familiaris	EEIVES**S**FPLNAIE	PCVICQGRPKNGCIVHGKTGHLMACFTCAKKLKKRNKPCPVCRQPIQMIVLTYFP
E. caballus	EENVES**S**FPLNAIE	PCVICQGRPKNGCIVHGKTGHLMACFTCAKKLKKRNKPCPVCRQPIQMIVLTYFP
U. maritimus	EEVVES**S**FPLNAIE	PCVICQGRPKNGCIVHGRTGHLMACFTCAKKLKKRNKPCPVCRQPIQMIVLTYFP
I. tridecemlineatus	EESMES**S**FPLNAIE	PCVICQGRPKNGCIVHGKTGHLMSCFTCAKKLKKRNKPCPVCRQPIQMIVLTYFN
F. catus	EEIVEP**S**FPHNAIE	PCVICQGRPKNGCIVHGKTGHLMACFTCAKKLKKRNKPCPVCRQPIQMIVLTYFP
M. musculus	DESVES**S**FPLNAIE	PCVICQGRPKNGCIVHGKTGHLMSCFTCAKKLKKRNKPCPVCRQPIQMIVLTYFN
C. canadensis	EESMES**S**FPLNAVE	PCVICQGRPKNGCIVHGKTGHLMSCFTCAKKLKKRNKPCPVCRQPIQMIVLTYFN
E. europaeus	EESTEF**S**FPFNATE	PCVICQGRPKNGCIIHGKTGHLMACFTCAKKLKKRNKPCPVCRQPIQMIVLTYFP
O. garnettii	EESMEY**S**FPLSATE	PCVICQGRPKNGCIVHGRTGHLMACFTCAKKLKKRNKPCPVCRQPIQMIVLTYFP
T. syrichta	EDGAEA**S**FPLNAIE	PCVICQGRPKNGCIVHGKTGHLMACFTCARKLKKRNKPCPVCRQPIQMIVLTYFP
M. domestica	ERSME-**S**IPPSTVE	PCVICQGRPKNGCIVHGKTGHLMACFTCAKKLKKRNKPCPVCRQPIQMIVLTYFL
M. auratus	EESVES**G**FSLNAIE	PCVICQGRPKNGCIVHGKTGHLMSCFPCAKKLKKRNKPCPVCRQPIQMIVLTYFS
M. ochrogaster	EESMES**G**FSLNATE	PCVICQGRPKNGCIVHGKTGHLMSCFTCAKKLKKRNKPCPVCRQPIQMIVLTYFS
L. africana	EESVES**R**YRPIATE	PCVICQDRPKNGCIVHGKTGHLMACFTCAKKLKKRNKPCPVCRQPIQMIVLTYFL
O. anatinus	EENEDS**K**LPLSTIE	PCVICQSRPKNGCILHGRTGHLMACFTCAKKLKKRNKPCPVCRQPIQMIVLTYFS
**Other animal classes**		
A. mississippiensis	EENTES**S**LPITSIE	PCVICQSRPKNGCIVHGKTGHLMSCFTCARKLKKRNKPCPVCRQPIQMIVLTYFG
A. aestiva	EESMES**S**LPVTCVE	PCVICQSRPKNGCIVHGKTGHLMSCFTCARKLKKRNKPCPVCRQPIQMIVLTYFS
X. laevis	KESMES**S**LPLTSID	PCVICQTRPKNGCIVHGRTGHLMACYTCAKKLKKRNKPCPVCREPIQMIVLTYFS
P. sinensis	EETMEC**S**LPLSSIE	PCVICQSRPKNGCIVHGKTGHLMSCFTCARKLKKRNKPCPVCRQPIQMIVQTYFS
P. nattereri	FNSLEA**S**LPTTCLE	PCVICQSRPKNGCIVHGRTGHLMACYTCAKKLKNRNKLCPVCREPISVIVLTYVS
M. fulvius	EEILEV**G**QPVSIVE	PCVICQTRPKNGCIVHGRTGHLMSCFVCAKKLKKRNKPCPVCRQPIEMIVLTYFC
D. rerio	FNSLEA**C**LPATCLE	PCVICQSRPKNGCIVHGRTGHLMACYTCAKKLKNRNKLCPVCREPIQSVVLTYMS

Supplementary Figure 6 Sequence alignment of MDM2 RING domain from different animal classes, related to Figure 4a. Human MDM2 S429 and the corresponding residue in other animal classes are highlighted in bold and colored red. Residue numbers on top are from the human MDM2 sequence. The RING domain is indicated.

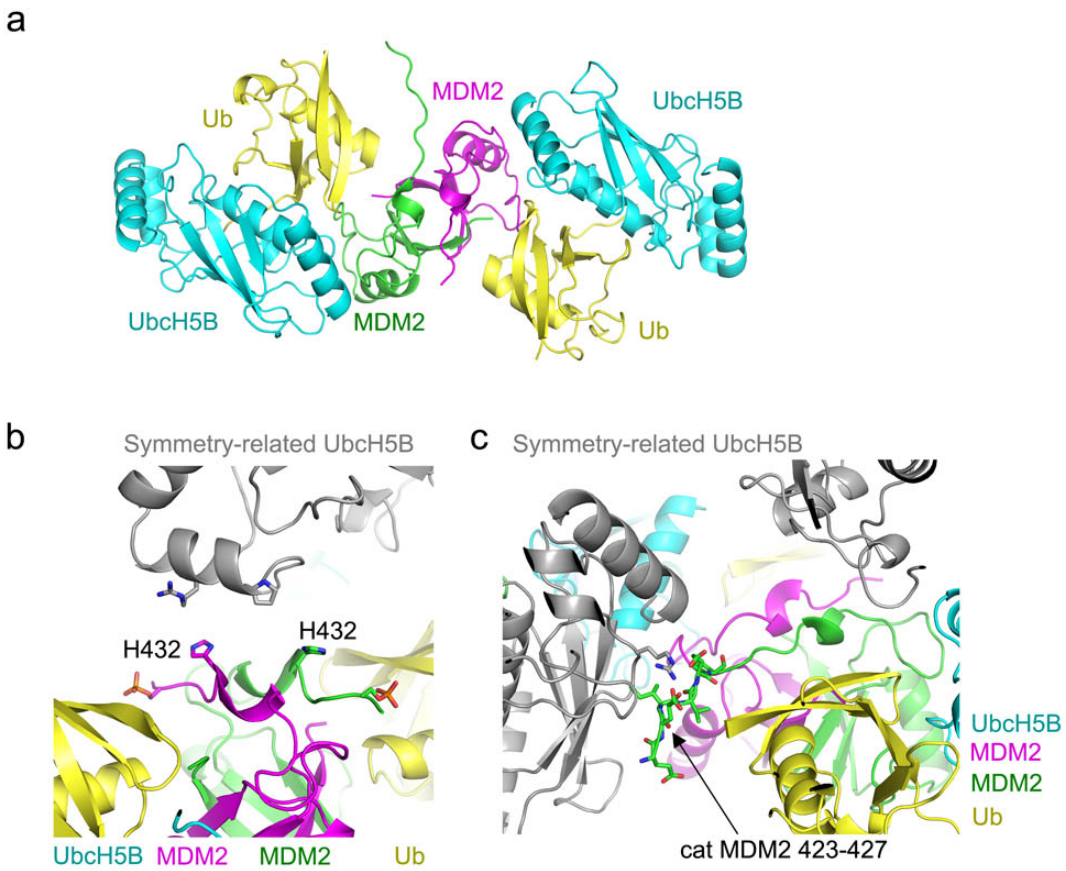

Supplementary Figure 7 Structure of cat MDM2-422–C-S429E-UbcH5B–Ub complex, related to Figure 4. (**a**) Cartoon representation of the complex. (**b**) Close up-view of crystal packing contacts in the cat MDM2-422–C-pS429-UbcH5B–Ub complex. H432 is unique to cat MDM2 (L432 in human MDM2) and packs against a symmetry-related UbcH5B molecule. (**c**) Close up-view of crystal packing contacts in the cat MDM2-422–C-S429E-UbcH5B–Ub complex. Cat MDM2's residues 423–427 pack against a symmetry-related UbcH5B molecule. All coloring is as described in **Figure 2**. Symmetry-related molecules are in gray.

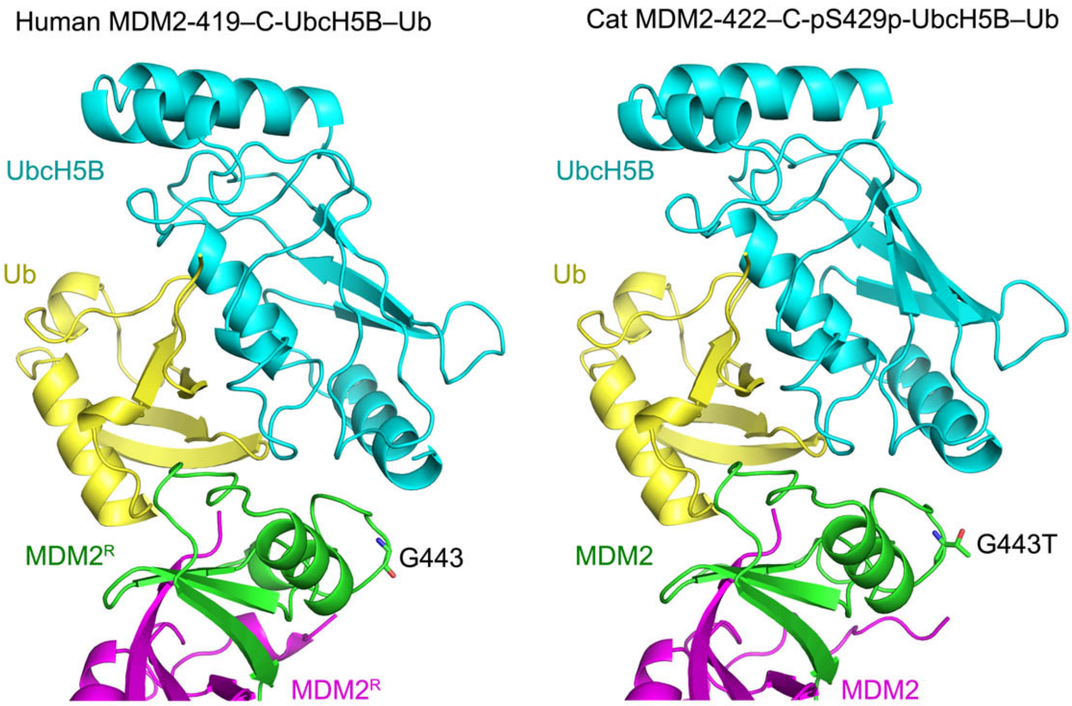

Supplementary Figure 8 Comparison of the structures of human MDM2-419–C-UbcH5B–Ub (left) and cat MDM2-422-C–pS429-UbcH5B–Ub (right) complexes showing the location of the G443T substitution in cat MDM2 RING domain. G443T sits at the periphery of the UbcH5B binding site and does not engage in UbcH5B binding or alter the structural fold of the RING domain. All coloring is as described in **Figure 2**.

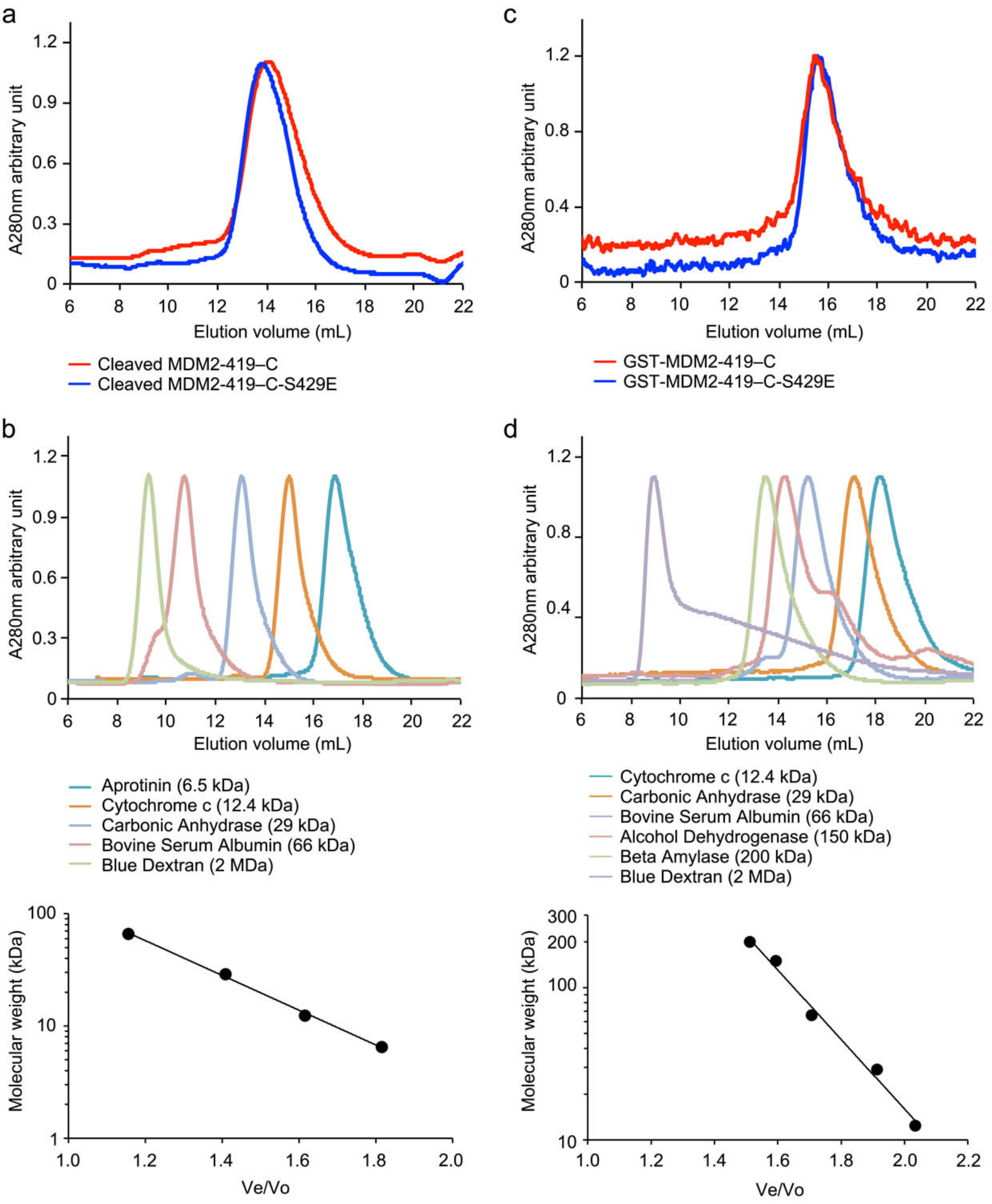

Supplementary Figure 9 Size-exclusion chromatography elution profiles of dimeric MDM2 RING domain constructs. (**a**) Superdex 75 10/300 elution profile of purified cleaved MDM2-419–C (red line) and cleaved MDM2-419–C-S429E (blue line). (**b**) Superdex 75 10/300 elution profile of indicated protein standards (top panel) and a semi-log plot of molecular weights of protein standards versus elution volume (Ve)/void volume (Vo) (bottom panel). Cleaved MDM2-419–C and cleaved MDM2-419–C-S429E have

estimated molecular weights of 19 and 20 kDa, respectively, consistent with a dimer (theoretical molecular weight of 17 kDa). (**c**) Superdex 200 10/300 elution profile of purified GST-MDM2-419–C (red line) and GST-MDM2-419–C-S429E (blue line). (**d**) Superdex 200 10/300 elution profile of indicated protein standards (top panel) and a semi-log plot of molecular weights of protein standards versus elution volume (Ve)/void volume (Vo) (bottom panel). GST-MDM2-419–C and GST-MDM2-419–C-S429E have estimated molecular weights of 65 kDa, consistent with a dimer (theoretical molecular weight of 70 kDa).

Figure 6a

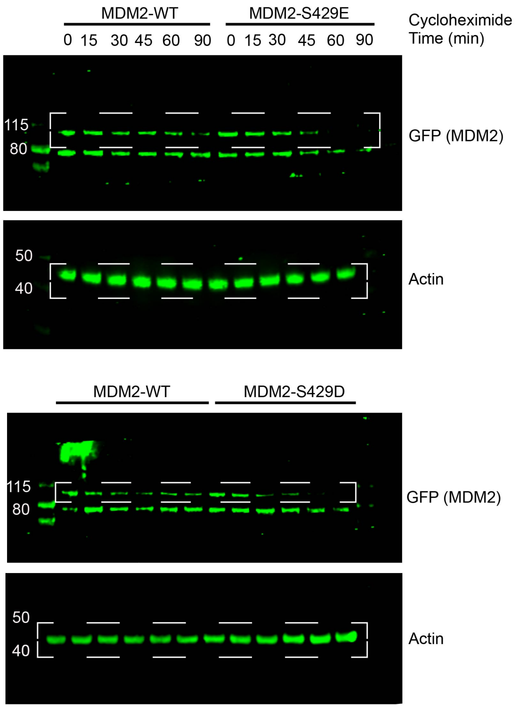

Figure 6b

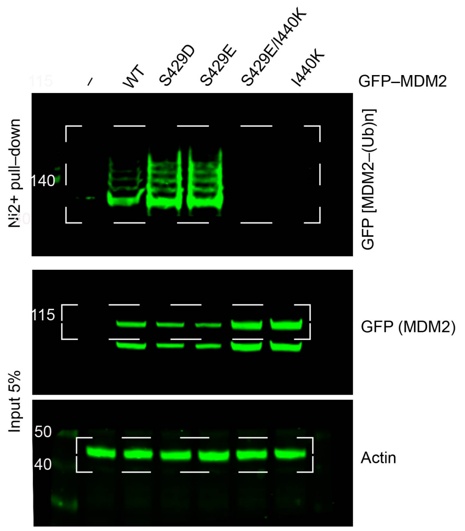

Figure 6c

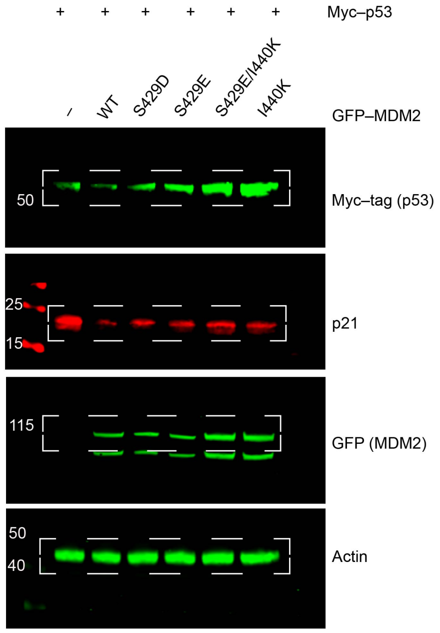

Figure 6d

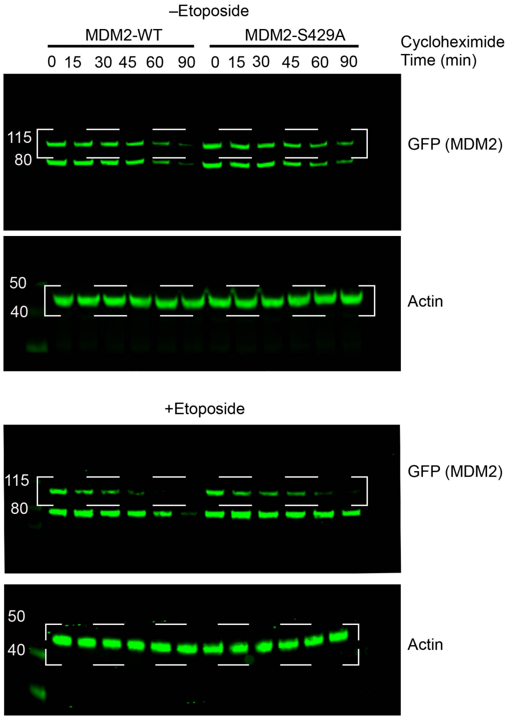

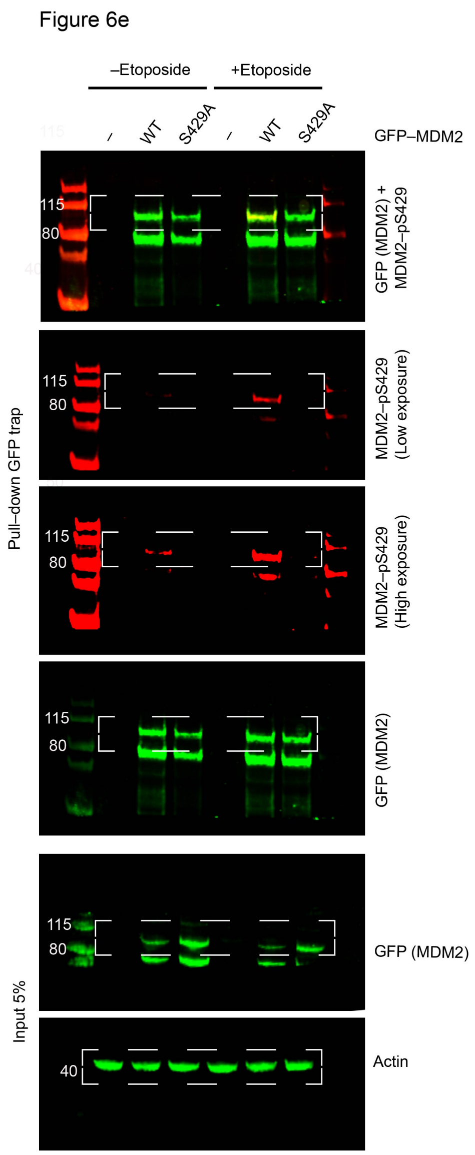

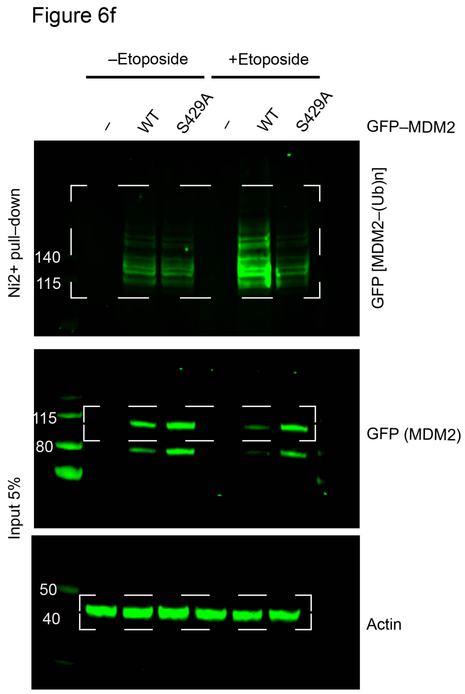

Supplementary Figure 10 Unprocessed images of immunoblots. Unprocessed images of scanned immunoblots as shown in **Figure 6** are provided. Regions that are presented in the main figures are outlined with a dashed grey rectangle.
