# Supplementary material for: Structural basis for DNA damage-induced phosphoregulation of MDM2 RING domain
Source: Nat Commun. 2020 Apr 29;11:2094. doi: 10.1038/s41467-020-15783-y (PMC7190642; doi:10.1038/s41467-020-15783-y)
Supplement: Supplementary file 3 — Reporting Summary [file 41467_2020_15783_MOESM3_ESM.pdf]

nature research

Corresponding author(s): Danny T Huang
Last updated by author(s): Mar 13, 2020

Reporting Summary

Nature Research wishes to improve the reproducibility of the work that we publish. This form provides structure for consistency and transparency in reporting. For further information on Nature Research policies, see Authors & Referees and the Editorial Policy Checklist.

Statistics

For all statistical analyses, confirm that the following items are present in the figure legend, table legend, main text, or Methods section.

n/a	Confirmed	
☐	☒	The exact sample size (*n*) for each experimental group/condition, given as a discrete number and unit of measurement
☐	☒	A statement on whether measurements were taken from distinct samples or whether the same sample was measured repeatedly
☒	☐	The statistical test(s) used AND whether they are one- or two-sided<br>*Only common tests should be described solely by name; describe more complex techniques in the Methods section.*
☒	☐	A description of all covariates tested
☒	☐	A description of any assumptions or corrections, such as tests of normality and adjustment for multiple comparisons
☐	☒	A full description of the statistical parameters including central tendency (e.g. means) or other basic estimates (e.g. regression coefficient) AND variation (e.g. standard deviation) or associated estimates of uncertainty (e.g. confidence intervals)
☒	☐	For null hypothesis testing, the test statistic (e.g. *F*, *t*, *r*) with confidence intervals, effect sizes, degrees of freedom and *P* value noted<br>*Give P values as exact values whenever suitable.*
☒	☐	For Bayesian analysis, information on the choice of priors and Markov chain Monte Carlo settings
☒	☐	For hierarchical and complex designs, identification of the appropriate level for tests and full reporting of outcomes
☒	☐	Estimates of effect sizes (e.g. Cohen's *d*, Pearson's *r*), indicating how they were calculated

Our web collection on statistics for biologists contains articles on many of the points above.

Software and code

Policy information about availability of computer code

Data collection	X-ray data collection was carried out at beamlines I03, I04 and I04-1 at Diamond Light Source (UK). Mass spectrometry data were acquired on a Orbitrap Q-Exactive HF mass spectrometer.
Data analysis	X-ray datasets were processed by XDS pipeline and reduced with fast_dp package or XIA2 package. Initial phasing was done by molecular replacement with PHASER and the atomic models were built with COOT and refined in PHENIX and REFMAC. Image Studio Lite 5.2 (LI-COR Biosciences) was used for quantification. Prism 8 was used to generate plots. Pymol was used to generate structural figures. Mass spectrometry data were processed using XCalibur and MaxQuant software and searched with Andromeda search engine.

For manuscripts utilizing custom algorithms or software that are central to the research but not yet described in published literature, software must be made available to editors/reviewers. We strongly encourage code deposition in a community repository (e.g. GitHub). See the Nature Research guidelines for submitting code & software for further information.

Data

Policy information about availability of data

All manuscripts must include a data availability statement. This statement should provide the following information, where applicable:

- Accession codes, unique identifiers, or web links for publicly available datasets
- A list of figures that have associated raw data
- A description of any restrictions on data availability

Atomic coordinates and structure factors are deposited in the Protein Data Bank with accession codes of 6SQO (human MDM2-419–C-UbcH5B–Ub complex), 6SQS (cat MDM2-422–C-pS429-UbcH5B–Ub complex), 6SQR (cat MDM2-422–C-S429E-UbcH5B–Ub complex), and 6SQP (cat MDM2-422–C-S429E). Raw data for Figures 1a, 1c and 1e are provided in Supplementary Figure 3, raw data for Figures 3f, 4b, 4i, 4k, 4m, 5f, 5h and 5j are provided in Supplementary Figure 5 and raw data for Figure 6 are provided in Supplementary Figure 10.

Field-specific reporting

Please select the one below that is the best fit for your research. If you are not sure, read the appropriate sections before making your selection.

☒ Life sciences ☐ Behavioural & social sciences ☐ Ecological, evolutionary & environmental sciences

For a reference copy of the document with all sections, see nature.com/documents/nr-reporting-summary-flat.pdf

Life sciences study design

All studies must disclose on these points even when the disclosure is negative.

Sample size	The study involves U2OS cells and in vitro experiments. For both in vitro and cell-based experiments, the sample size varied between 2-6.
Data exclusions	No data were excluded
Replication	Three independent reactions were performed for all activity assays presented in the manuscript. For all cell-based studies, experiment were performed at least three times. All attempts were successful
Randomization	There was no randomization in this study. The sample size was not high enough to effect the final outcome with non-randomized experimental set ups.
Blinding	There was no blinding in this study. The experimental set ups unsuitable for blinding.

Reporting for specific materials, systems and methods

We require information from authors about some types of materials, experimental systems and methods used in many studies. Here, indicate whether each material, system or method listed is relevant to your study. If you are not sure if a list item applies to your research, read the appropriate section before selecting a response.

Materials & experimental systems

n/a	Involved in the study
☐	☒ Antibodies
☐	☒ Eukaryotic cell lines
☒	☐ Palaeontology
☒	☐ Animals and other organisms
☒	☐ Human research participants
☒	☐ Clinical data

Methods

n/a	Involved in the study
☒	☐ ChIP-seq
☒	☐ Flow cytometry
☒	☐ MRI-based neuroimaging

Antibodies

Antibodies used	The primary antibodies used in this study include mouse anti-GFP (Santa Cruz Biotechnology, cat. no. sc-81045, clone no. A00185, Lot no. B1417, 1:1000 for western blot), customized rabbit anti-MDM2-pS429 (Eurogentec, 1:1000 for western blot), mouse anti-Myc tag (Cell Signaling Technology, cat. no. 2276, clone no. 9B11, Lot no. 24, 1:1,000 for western blot), rabbit anti-p21 (Cell Signaling Technology, cat no. 2947, clone no. 12D1, Lot no. 10, 1:1000 for Western blot) and goat anti-Actin (Santa Cruz Biotechnology, cat. no. sc-1616, Clone no. I-19, Lot no. G1615, 1:1000 for western blot).<br>The secondary antibodies used were goat anti-mouse IRDye 800CW (LI-COR Biosciences, cat no. 925-32210, Lot no. C61012-06, 1:15000 for Western blot), goat anti-rabbit IRDye 680LT (LI-COR Biosciences, cat no. 925-68021, Lot no. C80425-02, 1:20000 for Western blot) and donkey anti-goat IRDye 800CW (LI-COR Biosciences, cat no. 925-32214, Lot no. C50330-07, 1:15000 for Western blot)
Validation	mouse anti-GFP and goat anti-Actin: validation available on Santa Cruz Biotechnology website (https://www.scbt.com/p/gfp-antibody-a00185-01; https://www.scbt.com/p/actin-antibody-i-19?requestFrom=search)<br>customized rabbit anti-MDM2-pS429: validated in our study (Supplementary Figure 2)<br>mouse anti-Myc tag and rabbit anti-p21: validation available on Cell Signaling Technology website (https://www.cellsignal.co.uk/products/primary-antibodies/myc-tag-9b11-mouse-mab/2276; https://www.cellsignal.co.uk/products/primary-antibodies/p21-waf1-cip1-12d1-rabbit-mab/2947)<br>goat anti-mouse IRDye 800CW, goat anti-rabbit IRDye 680LT and donkey anti-goat IRDye 800CW: validation available on LI-COR Biosciences website (https://www.licor.com/bio/reagents/irdye-800cw-goat-anti-mouse-igg-secondary-antibody; https://www.licor.com/bio/reagents/irdye-680lt-goat-anti-rabbit-igg-secondary-antibody; https://www.licor.com/bio/reagents/irdye-800cw-donkey-anti-goat-igg-secondary-antibody)

Eukaryotic cell lines

Policy information about cell lines

Cell line source(s)	U2OS cells from ATCC
Authentication	In-house STR profiling using GenePrint 10 System (Promega)
Mycoplasma contamination	All cell lines are negative for mycoplasma contamination
Commonly misidentified lines (See ICLAC register)	No mis-identified cell line was used
